# The relationship between physical exercise and sleep procrastination in college students: the chain mediating role of self-control and time management tendencies

**DOI:** 10.3389/fpsyg.2026.1838723

**Published:** 2026-06-18

**Authors:** Jianhua Bu, Yikun Yin, Liang Yu

**Affiliations:** 1 College of Physical Education, Qiqihar University, Qiqihar, Heilongjiang, China; 2 Sport Science School, Beijing Sport University, Beijing, China; 3 School of Physical Education, Jining University, Jining, China

**Keywords:** bedtime procrastination, college students, physical exercise, self-control, time management tendency

## Abstract

**Objective:**

This study aims to investigate the direct effect of physical exercise on bedtime procrastination among college students, as well as the mediating roles of self-control and time management tendencies.

**Methods:**

A total of 1,069 college students from four universities were surveyed using an online questionnaire. The instruments included the Physical Activity Rating Scale (PARS-3), the Bedtime Procrastination Scale (BPS), the Self-Control Scale (SCS), and the Time Management Disposition Scale (TMD).

**Results:**

(1) Physical exercise was significantly negatively correlated with bedtime procrastination (*p* < 0.01) and significantly positively correlated with both self-control and time management tendencies (*p* < 0.01). Self-control and time management tendencies were also positively correlated with each other (*p* < 0.01), and both were negatively correlated with bedtime procrastination (*p* < 0.01). (2) Physical exercise had a significant negative predictive effect on bedtime procrastination (*β* = −0.158, *t* = −5.229, *p* < 0.01). Self-control and time management tendencies each played independent mediating roles in the relationship between physical exercise and bedtime procrastination. (3) A significant chain mediation pattern was observed, suggesting that physical exercise was indirectly associated with bedtime procrastination via self-control and time management tendencies (*β* = 0.058, *t* = 3.014, *p* < 0.01).

**Conclusion:**

Physical exercise significantly negatively predicts bedtime procrastination among college students. Self-control and time management tendencies serve as significant mediators in this relationship, forming a chain mediation pathway: Physical Exercise → Self-Control → Time Management Tendency → Bedtime Procrastination. These findings provide a theoretical basis for developing targeted behavioural interventions to improve sleep habits among university students.

## 1 Introduction

With the advent of the “digital-intelligent era” the use of electronic devices such as computers and mobile phones has become increasingly widespread (De Araújo et al., 2018). Young people now spend substantial amounts of time on computers, smartphones, tablets, and televisions, and this trend is particularly pronounced among college students, who constitute a significant segment of the youth population (Wang et al., 2019). College students’ daily lives are deeply integrated with digital devices, and the use of electronic products before bedtime is particularly widespread. According to the *China Sleep Research Report* (2024), 83.44% of college students use electronic devices before sleep, and sleep-related problems have become a serious and prevalent health concern among this population (Wang and Yue, 2024). Bedtime procrastination (BtP) refers to the behaviour in which individuals fail to go to bed or fall asleep at the intended time despite the absence of external constraints. A survey study reported a prevalence of 10.5% for sleep procrastination among Chinese college students (Zhang et al., 2025), indicating that it has become a relatively common phenomenon in this population. Bedtime procrastination not only reduces sleep duration but also leads to a range of negative outcomes, including decreased learning efficiency, daytime sleepiness, and emotional problems such as depression and anxiety (Carlson et al., 2023), while further increasing the risk of chronic conditions such as metabolic syndrome (Kamphorst et al., 2018).

Sleep procrastination is not only a behavioural outcome of poor time management but is also closely associated with individuals’ self-regulation abilities (Zimmerman et al., 2024). Self-control refers to the capacity to consciously regulate and restrain one’s impulses, desires, emotions, and responses to external temptations to conform to social norms and pursue long-term goals (de Ridder et al., 2012). Self-control functions as a protective factor against sleep procrastination and is an effective predictor of its severity (Yunfei and Xinze, 2023); individuals with higher levels of self-control are less likely to develop severe bedtime procrastination (Hong et al., 2024). Time management tendency refers to individuals’ cognitive characteristics regarding their attitudes toward time and their planning and use of time. It manifests through specific behaviours related to the organization and use of time (Bizhong and Xiaojun, 2022). Time-management disposition is negatively associated with bedtime procrastination; individuals with stronger time management abilities are better able to structure their daily routines and thus reduce procrastination at bedtime (Herzog-Krzywoszanska et al., 2024). Although previous studies have separately examined the roles of self-control and time-management disposition in bedtime procrastination, little research has explored their potential sequential relationship in the association between physical exercise and bedtime procrastination, particularly among Chinese college students. As a positive, health-enhancing lifestyle practice, physical exercise promotes both physical and mental well-being, improves behavioural habits, and enhances overall quality of life (Roychowdhury, 2020). The present study examined whether physical exercise was associated with bedtime procrastination through time-management disposition and self-regulatory processes among college students. The findings aim to enrich the behavioural mechanism model of bedtime procrastination and provide theoretical support and practical guidance for psychological interventions and health education programs in higher education institutions.

## 2 Literature review

### 2.1 Physical exercise and bedtime procrastination

As a positive lifestyle practice and behavioural intervention, physical exercise has been widely recognized for its substantial benefits to physical and mental health. It has been shown to effectively improve sleep quality through both physiological and psychological pathways (Arc-Chagnaud et al., 2024; Lang et al., 2022). Physical exercise promotes the release of neurotransmitters such as endorphins and serotonin, reduces the disruptive effects of somatic discomfort and psychological stress on sleep, accelerates sleep onset, and increases the duration of deep sleep (Schoenfeld and Swanson, 2021; Alnawwar et al., 2023). Moreover, exercise helps strengthen daily routines and structure, fosters regular sleep–wake habits, and enhances individuals’ time awareness and self-discipline regarding their schedules (Min et al., 2021). Research has demonstrated that engaging in 920 MET·min of physical activity per week can significantly improve sleep quality (Wang H. et al., 2025), while consistent exercise participation can alter daily routines and reduce bedtime procrastination among college students (Wang Y. et al., 2025). Additional findings indicate that both high-intensity interval training and weekly continuous aerobic exercise can significantly reduce sleep onset latency, increase total sleep duration, and improve sleep quality, thereby optimizing sleep behaviours in college populations (Min et al., 2021). Therefore, Hypothesis H1 is proposed: Physical exercise negatively predicts bedtime procrastination among college students.

### 2.2 Mediating role of self-control between physical exercise and bedtime procrastination

Self-control is regarded as an important psychological factor influencing bedtime procrastination (Yiming et al., 2020). According to the self-regulation resource theory, self-regulation depends on a limited pool of psychological resources that can become temporarily depleted after excessive use, and such depletion is significantly positively associated with bedtime procrastination (Kamphorst et al., 2018). For instance, repeatedly suppressing impulses, such as snacking or consuming high-calorie beverages during the day, may deplete individuals’ limited self-regulatory resources, thereby diminishing their ability to resist the temptation to stay up late and increasing the likelihood of bedtime procrastination (Muraven and Baumeister, 2000). Adequate self-control contributes to better sleep quality and longer sleep duration, while enhanced self-control helps foster regular, healthy sleep habits (Guarana et al., 2021). Research has shown that self-control can predict bedtime procrastination: higher levels of self-control are associated with improved sleep quality, whereas weaker self-control tends to result in bedtime procrastination and insufficient sleep, ultimately affecting physical and mental health (Yunfei and Xinze, 2023; Ling et al., 2024). Empirical studies have further demonstrated that physical exercise significantly enhances self-control by increasing functional activation in the prefrontal cortex, thereby improving self-regulation, executive function, and emotional regulation capacities (Zhao et al., 2023; Liu et al., 2024). Therefore, Hypothesis H2 is proposed: Self-control mediates the relationship between physical exercise and bedtime procrastination.

### 2.3 Mediating role of time management tendency between physical exercise and bedtime procrastination

Physical exercise is not only a behaviour that promotes physical health but also plays an important regulatory role at the psychological level. Research has shown that physical exercise exerts a positive influence on college students’ time management tendencies (Fen et al., 2011), likely because engaging in exercise requires individuals to schedule time, set plans, and adhere to routines-processes that implicitly cultivate and strengthen time awareness, prioritization skills, and behavioural self-discipline (Kundakcı et al., 2024; Boat and Cooper, 2019). Similarly, a tendency towards time management has been found to significantly and negatively predict procrastination behaviour, meaning that individuals with stronger time management abilities tend to show lower levels of procrastination (Yanhui, 2014). As a specific form of procrastination, bedtime procrastination is also significantly affected by time-management tendencies (Kroese et al., 2014). Time-management tendency negatively predicts bedtime procrastination, suggesting that it plays an important role in regulating self-control-related behaviours (Magalhães et al., 2020). In addition, regular physical activity is often accompanied by more structured routines-for example, fixed morning exercises or after-class workouts-which further reinforce rhythm and structure in daily life. Such behavioural patterns can facilitate the development of reasonable time-allocation strategies and enhance overall time-management tendencies (Arlinghaus and Johnston, 2019). Among college students, a tendency toward time management is closely related to exercise habits; those with more consistent exercise routines tend to exhibit stronger time-management abilities (Fen et al., 2011). Therefore, Hypothesis H3 is proposed: Time management tendency mediates the relationship between physical exercise and bedtime procrastination.

### 2.4 The chain mediating role of self-control and time management tendency in the relationship between physical exercise and bedtime procrastination

Both self-control and time-management tendencies are important factors influencing bedtime procrastination among college students, and each plays a significant mediating role in the relationship between physical exercise and bedtime procrastination (Yiming et al., 2020; Fen et al., 2011). Physical exercise not only enhances individuals’ self-control but also significantly improves their time management tendencies. Although self-control and time management represent distinct psychological and behavioural characteristics, they are functionally interconnected. Research indicates that self-control can effectively predict time-management tendencies; individuals with weaker self-control have more difficulty regulating and restraining their thoughts and behaviours (Aeon et al., 2021), which, in turn, leads to poorer time-management tendencies and a higher likelihood of bedtime procrastination. Taken together, self-control may constitute an essential internal foundation for the development of time management tendencies. While significant associations have been observed among physical exercise, self-control, time management tendency, and bedtime procrastination, systematic research on the internal linkages and interaction mechanisms among these four variables remains limited. Therefore, the present study takes physical exercise as the independent variable and bedtime procrastination as the dependent variable, with self-control and time-management tendencies as chain mediators, to examine the underlying pathways linking physical exercise and bedtime procrastination. Hypothesis H4 is thus proposed: Self-control and time management tendency jointly serve as chain mediators in the relationship between physical exercise and bedtime procrastination among college students.

**Figure 1:**
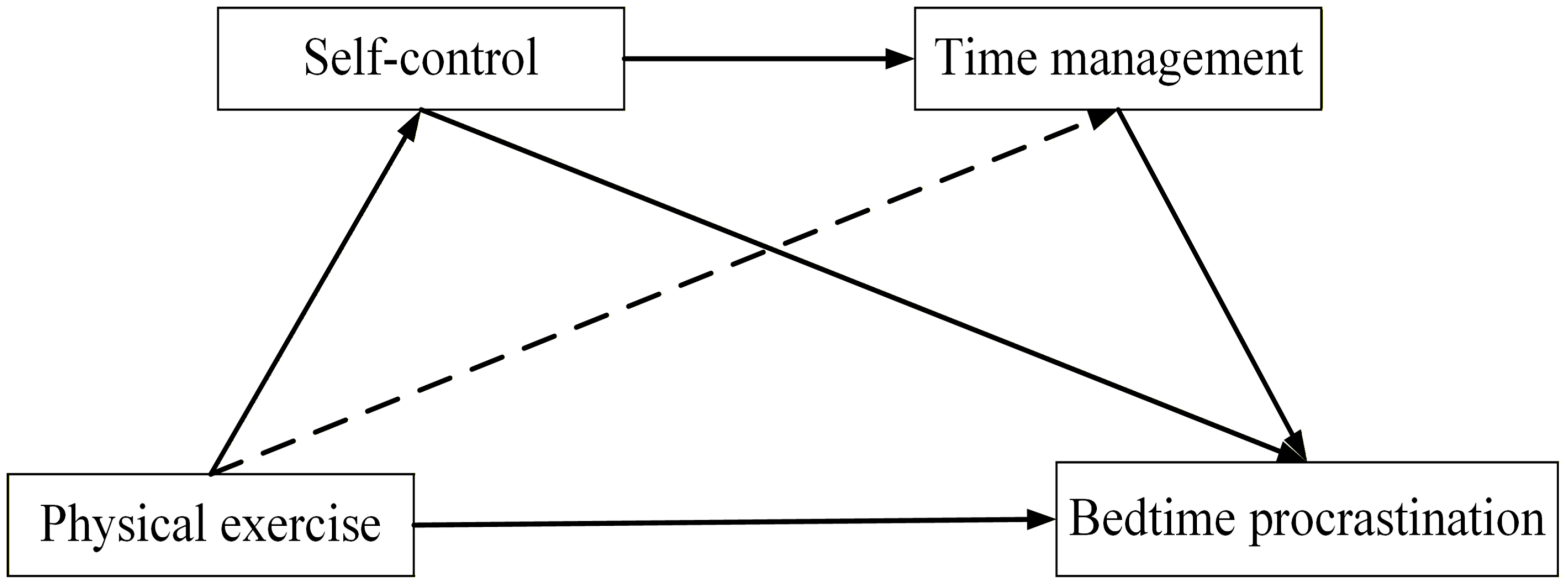
Hypothetical model of the influence of physical exercise on bedtime procrastination.

Based on this, the study will explore the impact of physical exercise on sleep procrastination behaviour among college students, reveal the roles of self-control and time-management tendencies in this influence, and construct a chain mediation model (Figure 1).

## 3 Methods

### 3.1 Data and sample

From October 2024 to October 2025, an online questionnaire survey was conducted using cluster sampling to recruit college students from four universities located in Shandong, Fujian, Shaanxi, and Guizhou. Before data collection, physical education teachers at each participating institution received standardized training and instructions that clarified the survey objectives, questionnaire structure, and response criteria to ensure scientific rigour and consistency throughout the investigation. During the formal data collection process, the questionnaires were distributed electronically via the “Wen Juan Xing” platform. All students completed the survey anonymously under the unified organization of their teachers before physical education classes, ensuring both procedural standardization and data confidentiality. A total of 1,125 questionnaires were distributed, of which 1,106 were returned, yielding a response rate of 98.3%. After excluding invalid responses, 1,069 valid questionnaires were retained for analysis, corresponding to an effective response rate of 96.7%. Based on answering time (≥5 min, the average minimum required), completeness (excluding responses with identical answers to five consecutive items), and logical consistency, 37 invalid questionnaires were removed. Ultimately, 1,069 valid questionnaires were included in the analysis. Among the respondents, 320 were male, and 749 were female, with an average age of 19.06 ± 1.17 years. All participants provided informed consent, and the study was approved by the Biomedical Ethics Committee of Jining University (Approval No.: 2024JNXYLL-034).

### 3.2 Research tools

#### 3.2.1 Physical activity rating scale-3 (PARS-3)

The scale revised by Liang (1994) was used to assess physical activity among college students across three dimensions: exercise intensity, exercise duration, and exercise frequency. A 5-point Likert scoring method was adopted, with exercise intensity scored from 1 to 5 points, exercise duration from 0 to 4 points, and exercise frequency from 1 to 5 points. The total physical activity score was calculated as: Physical activity volume = intensity × duration × frequency, yielding a score ranging from 0 to 100, where higher scores indicate greater physical activity. To enhance comparability among dimensions and eliminate the influence of differing units, the physical activity scores were standardized using Z-scores.

#### 3.2.2 Self-control scale (SCS)

The scale revised by Tan and Guo (2008) was adopted. It consists of 19 items covering five dimensions: impulse control, healthy habits, restrained entertainment, focused work, and resistance to temptation. A 5-point Likert scoring format was used (1 = completely inconsistent, 5 = completely consistent). Items 2, 3, 4, 6, 7, 8, 9, 10, 12, 13, 15, 16, 17, 18, and 19 are reverse-scored. The total score ranges from 19 to 95, with higher scores indicating stronger self-control. In the present study, the Cronbach’s *α* coefficient for the scale was 0.91.

#### 3.2.3 Time management disposition scale (TMD)

The Time Management Disposition Scale for adolescents, developed by Xiting and Zhijie (2001), was used. The scale consists of 44 items across three dimensions: sense of time value, view of time monitoring, and sense of time efficacy. A 5-point Likert scale was used (1 = completely inconsistent, 5 = completely consistent). Items 9, 17, 27, 30, and 41 are reverse-scored. The total score is 220, with higher scores indicating a stronger time management disposition. In this study, the Cronbach’s *α* coefficient for the scale was 0.95.

#### 3.2.4 Bedtime procrastination scale (BPS)

The Chinese version of the BPS developed by Xiao-Han et al. (2021) was used. The scale consists of 9 items, scored on a 5-point Likert scale (1 = completely inconsistent, 5 = completely consistent). Items 2, 3, 7, and 9 are reverse-scored. The total score is divided by 9 to obtain the average score, with higher averages indicating more severe bedtime procrastination. In the present study, the Cronbach’s *α* coefficient for the scale was 0.74.

### 3.3 Statistical analysis

SPSS 26.0 was used for data sorting and statistical analysis. Categorical variables were described as frequencies and percentages. The Kolmogorov–Smirnov (K–S) test, together with skewness and kurtosis values, was adopted to evaluate the normality of continuous variables. A non-significant result (*p* > 0.05) in the K–S test was considered indicative of approximate normality, supplemented by skewness and kurtosis values within ±2.0. Pearson correlation analysis was conducted for normally distributed data, while Spearman’s rank correlation analysis was utilized for non-normally distributed data. PROCESS 3.5 was employed to conduct regression-based mediation analysis to examine the chain-mediated effects among physical exercise, self-control, time-management disposition, and bedtime procrastination. The mediation effects were tested using the Bootstrap method, with 5,000 resamples to estimate 95% confidence intervals for the indirect effects. Mediation was considered significant when the confidence interval did not include zero. To assess the robustness of the proposed model and address potential model equivalence, an alternative sequential mediation model was tested by reversing the order of self-control and time management disposition. All statistical results were reported as mean ± standard deviation (M ± SD), and a significance level of *p* < 0.05 was adopted.

## 4 Results

### 4.1 Common method bias test

Because all variables in this study were measured using self-reported questionnaires, potential common method bias may be present. To examine this issue, Harman’s single-factor test was conducted by performing an unrotated exploratory factor analysis on all measurement items. The results showed that 15 factors had eigenvalues greater than 1, and the first factor accounted for 26.78% of the variance, which is well below the 40% threshold. These findings indicate that common method bias is not a serious concern in this study.

### 4.2 Descriptive statistics and correlation analysis of variables

Correlation analyses showed that physical exercise was positively associated with self-control (*r* = 0.092, *p* < 0.01) and time management disposition (*r* = 0.125, *p* < 0.01), and negatively associated with bedtime procrastination (*r* = −0.158, *p* < 0.01). Although these associations were statistically significant, the effect sizes were relatively small. Self-control was significantly and positively correlated with time management disposition (*r* = 0.647, *p* < 0.01), and both variables were significantly and negatively correlated with bedtime procrastination (*r* = −0.538 and −0.609, respectively, *p* < 0.01). These results indicate that college students who engage in higher levels of physical exercise tend to show stronger self-control, a better disposition toward time management, and lower levels of bedtime procrastination. Additionally, both self-control and time-management dispositions jointly predict bedtime procrastination, suggesting that they may mediate the relationship between physical exercise and bedtime procrastination (see Table 1).

**Table 1:** Means, standard deviations, and correlation coefficients of the study variables.

Variable	M	SD	Physical exercise	Self-control	Time management	Bedtime procrastination
Physical exercise	31.51	11.47	1			
Self-control	3.28	0.63	0.092^**^	1		
Time management	3.20	0.57	0.125^**^	0.647^**^	1	
Bedtime procrastination	3.15	0.39	−0.158^**^	−0.538^**^	−0.609^**^	1

**p < 0.01, *p < 0.05.

### 4.3 Mediating effects of physical exercise on bedtime procrastination

Significant correlations were found among physical exercise, self-control, time-management disposition, and bedtime procrastination, meeting the statistical prerequisites for conducting a mediation analysis. Based on the correlation results, regression analyses were performed with the former three variables as predictors and bedtime procrastination as the outcome variable. The detailed statistical results are presented in Table 2.

**Table 2:** The chain mediating model coefficients of self-control and time management disposition in the impact of physical exercise on bedtime procrastination.

Predictive variables	Self-control			Time management			Bedtime procrastination		
	*β*	SE	*t*	*β*	SE	*t*	*β*	SE	*t*
Physical exercise	0.0582	0.019	3.014^**^	0.037	0.013	2.823^**^	−0.158	0.012	−5.229^***^
Self-control				0.572	0.021	27.447^***^	−0.150	0.019	−7.981^***^
Time management							−0.301	0.021	−14.251^***^
*R* ^2^	0.008			0.423			0.413		
*F*	9.084^***^			391.061^***^			250.153^***^		

***p < 0.001, **p < 0.01, *p < 0.05.

Physical exercise had a significant negative predictive effect on bedtime procrastination (*β* = −0.158, *t* = −5.229, *p* < 0.001), accounting for 2.5% of the variance. When physical exercise, self-control, and time-management disposition were entered simultaneously into the regression equation, the effect of physical exercise on bedtime procrastination remained significant (*β* = −0.031, *t* = −3.403, *p* < 0.001). Physical exercise significantly and positively predicted self-control (*β* = 0.058, *t* = 3.014, *p* < 0.01) as well as time management disposition (*β* = 0.037, *t* = 2.823, *p* < 0.01). Self-control significantly and positively predicted time management disposition (*β* = 0.572, *t* = 27.447, *p* < 0.001), and significantly and negatively predicted bedtime procrastination (*β* = −0.150, *t* = −7.981, *p* < 0.001). Time management disposition also significantly and negatively predicted bedtime procrastination (*β* = −0.301, *t* = −14.251, *p* < 0.001). For explained variance (*R*^2^): Physical exercise accounted for 0.84% of the variance in self-control. Physical exercise and self-control jointly explained 42.32% of the variance in time management disposition, with standardized contributions of 6.59% and 64.12%, respectively. Physical exercise, self-control, and time management disposition together explained 41.34% of the variance in bedtime procrastination, with standardized contributions of 8.05% for physical exercise, 24.57% for self-control, and 44.04% for time management disposition.

**Figure 2:**
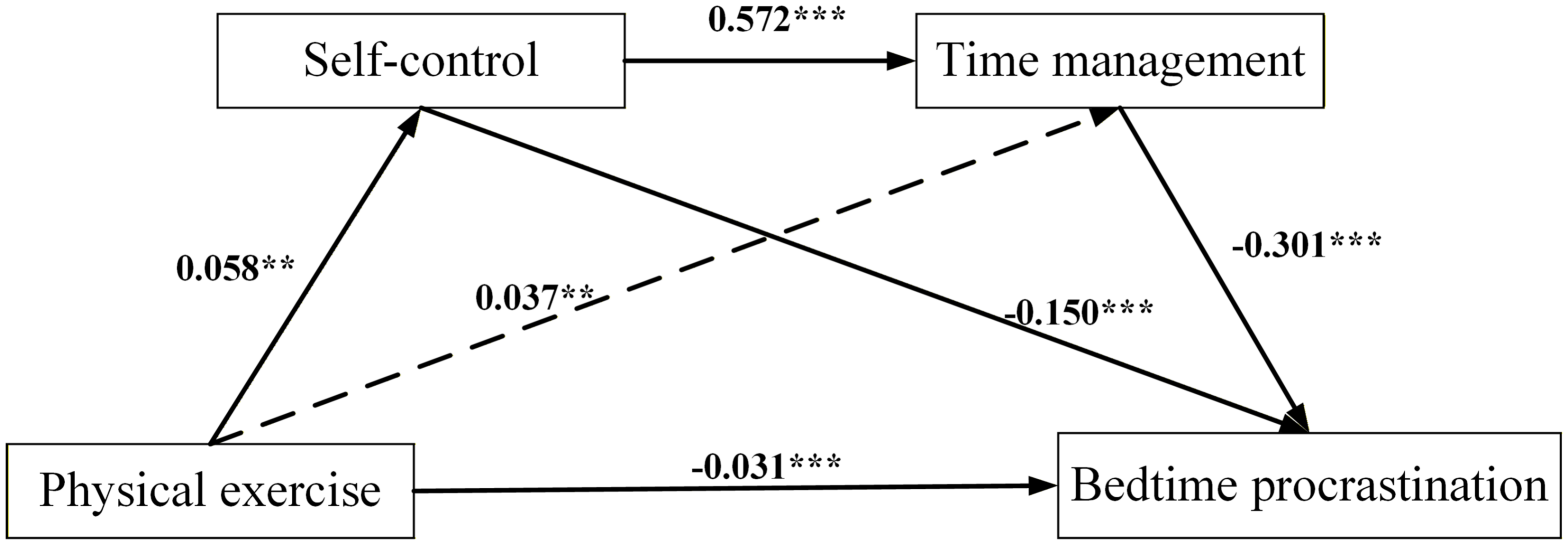
Structural path model of the effect of physical exercise on bedtime procrastination.

Following the mediation testing procedure proposed by Zhonglin et al. (2004), the mediation effects were examined. As shown in Table 3, both self-control and time-management tendency served as mediators in the relationship between physical exercise and bedtime procrastination. The total indirect effect was −0.0299, with a 95% bootstrap CI of [−0.0438, −0.0167]. Because the confidence interval did not include zero, the overall mediation effect was significant, accounting for 49.02% of the total effect (−0.0610). Three specific indirect pathways were identified. Physical exercise → self-control → bedtime procrastination: The indirect effect was −0.0087 (Bootstrap 95% CI [−0.0161, −0.0028]), indicating a significant mediation effect and accounting for 14.26% of the total effect. Physical exercise → time management tendency → bedtime procrastination: The indirect effect was −0.0112 (Bootstrap 95% CI [−0.0195, −0.0032]); because the interval did not contain zero, this path was significant, accounting for 18.36% of the total effect. Physical exercise → self-control → time management tendency → bedtime procrastination: The indirect effect was −0.0100 (Bootstrap 95% CI [−0.0167, −0.0036]), supporting a significant chain mediation effect and accounting for 16.39% of the total effect. Pairwise comparisons of the three indirect effects (C1, C2, C3) showed that all Bootstrap 95% CIs included zero, indicating no significant differences in the magnitudes of the indirect pathways. Figure 2 presents the chain mediation model, illustrating both the direct association between physical exercise and bedtime procrastination and the indirect pathways through self-control and time-management disposition.

**Table 3:** Chain mediation analysis of self-control and time management disposition.

Pathway	Effect	BootSE	BootLLCI	BootULCI	Relative effect proportion
Physical exercise → self-control → bedtime procrastination (Ind1)	−0.009	0.003	−0.016	−0.003	14.260
Physical exercise → time management → bedtime procrastination (Ind2)	−0.011	0.004	−0.020	−0.003	18.360
Physical exercise → self-control → time management → bedtime procrastination (Ind3)	−0.010	0.003	−0.017	−0.004	16.390
Total indirect effect	−0.030	0.007	−0.044	−0.017	49.020
Direct effect	−0.031	0.009	−0.049	−0.013	
Total effect	−0.061	0.012	−0.084	−0.038	
C1 (Ind1-Ind2)	0.003	0.006	−0.010	0.014	
C2 (Ind1-Ind3)	0.001	0.002	−0.003	0.005	
C3 (Ind2-Ind3)	−0.001	0.006	−0.012	0.010	

To further examine the robustness of the proposed model, an alternative chain mediation model was tested by reversing the order of self-control and time management disposition. The results indicated that the indirect effect through time management disposition remained significant. In contrast, the indirect effect through self-control alone was not significant, as the bootstrap confidence interval included zero. However, the chain mediation pathway (time management → self-control) remained significant. Furthermore, to control for potential confounding effects, gender and age were included as covariates in the mediation analysis. The overall pattern of results remained largely consistent, with the significant pathways preserved and the non-significant pathway remaining non-significant, supporting the robustness of the findings. The results of the alternative model and the covariate-adjusted model are provided in the Supplementary material.

## 5 Discussion

This study focuses on bedtime procrastination among college students and investigates the underlying mechanisms by which physical exercise influences this behaviour. By incorporating self-control and time-management tendencies as mediators, a chained mediation model was constructed and empirically tested. The results showed that the total effect of physical exercise on bedtime procrastination was −0.0610, with a direct impact of −0.0311 and a total indirect effect of −0.0299. All mediation effects, including the mediating roles of self-control and time management tendency, and their chained pathway, were statistically significant. These findings support the proposed chained mediation model and confirm all four hypotheses of this study.

### 5.1 Physical exercise and bedtime procrastination

This study found that physical exercise was significantly negatively associated with bedtime procrastination among college students, consistent with Hypothesis H1. This finding aligns with previous research (Wang Y. et al., 2025). From the perspective of the Theory of Planned Behaviour (TPB), behaviour is guided by behavioural intention, which is jointly influenced by attitudes, subjective norms, and perceived behavioural control (Campbell, 2010). Positive attitudes toward a behaviour and stronger perceived control are associated with a higher likelihood of behavioural engagement. Thus, when students view exercise favourably and perceive greater control over their sleep schedules, they may be more likely to engage in behaviours that support timely sleep. Physical exercise may be associated with more positive attitudes toward healthy routines, enhanced self-regulatory resources, and stronger perceived control over sleep-related behaviour, which may, in turn, be associated with lower levels of bedtime procrastination (Meng et al., 2024). Moreover, prior research suggests that exercise is associated with improvements in neurophysiological functioning, cognitive performance, emotional regulation, and circadian rhythm stability, all of which are related to better sleep outcomes (Mu et al., 2024; Shen et al., 2023). Moderate-intensity exercise has also been associated with reduced stress and anxiety, fewer pre-sleep emotional disturbances, and greater readiness for sleep, which may contribute to lower bedtime procrastination (Ye et al., 2022). Taken together, the findings of this study provide additional support for a negative association between physical exercise and bedtime procrastination.

### 5.2 The mediating role of self-control

The results of this study indicated that physical exercise was not only directly associated with bedtime procrastination but also indirectly associated with it via self-control, consistent with Hypothesis H2. This finding is consistent with previous research (Ye et al., 2025). According to the Self-Regulatory Failure Theory, procrastination is essentially a manifestation of self-regulatory failure (Rebetez et al., 2016). Bedtime procrastination falls into this category, as individuals often lack sufficient self-control to adhere to the long-term goal of going to bed on time when faced with the immediate rewards of browsing on mobile phones or other forms of entertainment. The Strength Model of Self-Control posits that self-control functions as a limited yet trainable psychological resource that supports goal-directed behaviour under conditions of temptation or conflict (Baumeister et al., 2007). Physical exercise may be associated with greater self-control resources, which may in turn be linked to fewer impulsive responses to nighttime temptations such as smartphone use and entertainment. Neuroimaging studies have shown that individuals who procrastinate exhibit reduced activation in the prefrontal cortex, particularly in regions associated with inhibitory control, working memory, and cognitive flexibility (Le Bouc and Pessiglione, 2022). Regular aerobic exercise has been associated with increased gray matter density and neuro-metabolic activity in the prefrontal cortex, as well as enhanced executive functioning (Chai et al., 2025), which may be related to problematic behaviours such as bedtime procrastination. Taken together, the findings suggest that self-control may be an important variable associated with the relationship between physical exercise and bedtime procrastination. However, it should be noted that the variance explained by physical exercise alone was relatively small, suggesting that it accounts for only a limited proportion of the variance in self-control.

### 5.3 The mediating role of time management disposition

The findings of this study showed that physical exercise was indirectly and negatively associated with bedtime procrastination via time management disposition among college students, consistent with prior research (Cao et al., 2018) and supporting Hypothesis H3. According to the Temporal Self-Regulation Theory (TST), engagement in health-related behaviours is influenced not only by behavioural intention but also by individuals’ time perspectives and self-regulation abilities (Sallis, 2010). Bedtime procrastination may reflect a state of self-regulatory imbalance under the conflict between immediate temptations and long-term goals. Individuals with a stronger time-management disposition typically have more structured daily routines, clearer goal orientations, and greater future-oriented awareness, which are associated with more effective time allocation and lower levels of sleep-onset delays. Physical exercise may be associated with better time-management skills and behavioural control, which in turn may be linked to improved time use and lower levels of bedtime procrastination during the pre-sleep period (Cao and Jiang, 2025). Taken together, the findings suggest that time-management disposition may be an important variable associated with the relationship between physical exercise and bedtime procrastination.

### 5.4 The chain mediating role of self-control and time management disposition

This study found that, in addition to their independent roles, time management disposition and self-control were observed as sequential mediators in the relationship between physical exercise and bedtime procrastination. Specifically, physical exercise was indirectly associated with bedtime procrastination through time management disposition and a subsequent pathway involving self-control. This finding is consistent with Hypothesis H4. Notably, the indirect effect through time management disposition remained statistically significant, whereas the indirect effect through self-control alone was not significant. According to self-regulation theory, individuals possess limited self-regulatory resources (Yiming et al., 2020). When substantial resources are consumed by cognitive demands or emotional regulation in other contexts, these reserves may become depleted, which may be associated with reduced self-control. This may make it more difficult to manage pre-sleep behaviours and may be related to delayed sleep onset, reduced sleep duration, and sleep-related problems, including insufficient sleep and fatigue (Exelmans and Van Den Bulck, 2018). Conversely, individuals with higher self-regulatory abilities typically demonstrate stronger cognitive control (Bandura, 1977), which is associated with better task prioritization, adaptive planning, and more effective time management. These individuals may be more likely to schedule study and rest periods in a structured manner, set clearer bedtime goals, and maintain more consistent behavioural monitoring, which may be associated with lower levels of pre-sleep smartphone use, more regular sleep routines, and lower levels of bedtime procrastination. Physical exercise has been associated with higher levels of self-control, as it is itself a structured, self-regulated behaviour (Kequn et al., 2025). Long-term participation in regular exercise has been linked to improved impulse control and interference inhibition under conditions of temptation or conflict. Taken together, these findings suggest that time management disposition plays a leading role, while self-control operates within a sequential pathway, in the relationship between physical exercise and bedtime procrastination.

Taken together, these findings suggest that time-management disposition may play a more central role than self-control in the relationship between physical exercise and bedtime procrastination. Compared with previous studies that primarily examined the independent roles of self-control or time-management disposition, the present study further explored their potential sequential relationship, thereby extending the understanding of the behavioural mechanisms underlying bedtime procrastination. In addition, the present study provides evidence from a large sample of Chinese college students, contributing to the understanding of bedtime procrastination within a specific cultural and educational context. Given the increased autonomy and lifestyle flexibility during university years, college students may be more vulnerable to bedtime procrastination. As a structured and sustainable health behaviour, physical exercise may help strengthen self-regulation and time-management awareness, suggesting that it may serve as a potential intervention strategy for reducing bedtime procrastination among college students.

## 6 Limitations and future directions

This study employed a cross-sectional design to examine the associations among physical exercise, self-control, time-management disposition, and bedtime procrastination. However, such a design does not allow for clear inferences about causal directions or temporal dynamics. Future studies may consider longitudinal designs to explore further the temporal relationships among physical exercise, self-regulatory abilities, and sleep-related behaviours. Additionally, all variables were measured using self-report questionnaires at a single time point, which may introduce common-method bias and social desirability bias. Although participants were informed that the survey was anonymous and confidential, and that there were no right or wrong answers to minimize potential response bias, method-related bias cannot be completely excluded. Moreover, Harman’s single-factor test has certain limitations and cannot fully rule out common method variance. Therefore, the observed associations may be inflated to some extent. Future research is encouraged to adopt multiple sources of data, objective measures, or longitudinal designs to reduce potential bias. In addition, with larger sample sizes, more robust statistical approaches, such as CFA marker-variable techniques or latent method factor modelling, are recommended to better control for method-related variance. Furthermore, the sample was drawn exclusively from four universities in China, which may limit the external validity and generalizability of the findings. Cultural and contextual factors, such as academic systems and lifestyle patterns, may influence the observed associations, and caution should be exercised when generalizing the results to other populations. In addition, the sample exhibited a gender imbalance, with a higher proportion of female participants. Although gender was included as a covariate and the results remained largely consistent, future studies should recruit more balanced samples to validate these findings further. The present findings suggest that the association between physical exercise and bedtime procrastination may involve multiple pathways, including the sequential role of self-control and time-management disposition. Future research could incorporate dynamic modelling approaches, such as multilevel modelling (MLM) or cross-lagged panel models (CLPM), to better understand these relationships.

## 7 Conclusion

Physical exercise, self-control, time management disposition, and bedtime procrastination among college students are significantly correlated with one another. Physical exercise positively predicts self-control and time-management dispositions, while negatively predicting bedtime procrastination. Self-control positively predicts time management disposition and negatively predicts bedtime procrastination. Time management disposition also negatively predicts bedtime procrastination. Both self-control and time-management dispositions independently mediate the relationship between physical exercise and bedtime procrastination. Furthermore, they jointly serve as sequential mediators in the relationship between physical exercise and bedtime procrastination among college students.

## Data Availability

The raw data supporting the conclusions of this article will be made available by the authors, without undue reservation.
