# Supplementary material for: The relationship between physical exercise and sleep procrastination in college students: the chain mediating role of self-control and time management tendencies
Source: Front Psychol. 2026 Jun 18;17:1838723. doi: 10.3389/fpsyg.2026.1838723 (PMC13322828; doi:10.3389/fpsyg.2026.1838723)
Supplement: Supplementary file 1 [file Table_1.DOCX]

**Supplementary Material：**

**The results of the alternative model（Physical exercise →time management tendency→ self-control → bedtime procrastination）**

**Fig. S1. Regression Coefficients for the Alternative Chain Mediation Model(Physical Exercise → Time Management → Self-Control → Bedtime Procrastination)**

**Table S1. Regression Coefficients for the Alternative Chain Mediation Model**

| Predictive variables | Time management | | | Self-control | | | bedtime procrastination | | |
| --- | --- | --- | --- | --- | --- | --- | --- | --- | --- |
|  | β | SE | t | β | SE | t | β | SE | t |
| Physical exercise | 0.071 | 0.017 | 4.110^***^ | 0.007 | 0.015 | 0.478 | -0.031 | 0.009 | -3.403^***^ |
| Self-control |  |  |  | 0.724 | 0.026 | 27.447^***^ | -0.301 | 0.021 | -14.251^***^ |
| Time management |  |  |  |  |  |  | -0.150 | 0.019 | -7.981^***^ |
| R² | 0.016 | | | 0.419 | | | 0.413 | | |
| F | 16.892^***^ | | | 384.402^***^ | | | 250.153^***^ | | |

**Table S2. Indirect Effects in the Alternative Chain Mediation Model**

| Pathway | Effect | BootSE | BootLLCI | BootULCI | Relative effect proportion |
| --- | --- | --- | --- | --- | --- |
| Physical exercise → Time management → Bedtime procrastination (Ind1) | -0.021 | 0.005 | -0.031 | -0.012 | 34.43 |
| Physical exercise → Self-control → Bedtime procrastination (Ind2) | -0.001 | 0.002 | -0.006 | 0.003 | 1.80 |
| Physical exercise → Time management → Self-control → Bedtime procrastination (Ind3) | -0.008 | 0.002 | -0.012 | -0.004 | 12.46 |
| Total Indirect Effect | -0.030 | 0.007 | -0.043 | -0.017 |  |
| Direct Effect | -0.031 | 0.009 | -0.049 | -0.013 |  |
| Total Effect | -0.061 | 0.012 | -0.084 | -0.038 |  |
| C1（Ind1-Ind2） | -0.020 | 0.006 | -0.033 | -0.008 |  |
| C2（Ind1-Ind3） | -0.014 | 0.005 | -0.024 | -0.005 |  |
| C3（Ind2-Ind3） | 0.006 | 0.003 | 0.001 | 0.012 |  |

**Table S3. Indirect Effects in the Covariate-Adjusted Mediation Model**

**(Age and Gender Controlled)**

| Pathway | Effect | BootSE | BootLLCI | BootULCI | Relative effect proportion |
| --- | --- | --- | --- | --- | --- |
| Physical exercise→Self-control→Bedtime procrastination（Ind1） | -0.009 | 0.003 | -0.016 | -0.003 | 14.260 |
| Physical exercise→Time management→Bedtime procrastination（Ind2） | -0.011 | 0.004 | -0.020 | -0.003 | 18.360 |
| Physical exercise→Self-control→Time management→Bedtime procrastination（Ind3） | -0.010 | 0.003 | -0.017 | -0.004 | 16.390 |
| Total Indirect Effect | -0.030 | 0.007 | -0.044 | -0.017 | 49.020 |
| Direct Effect | -0.031 | 0.009 | -0.049 | -0.013 |  |
| Total Effect | -0.061 | 0.012 | -0.084 | -0.038 |  |
| C1（Ind1-Ind2） | 0.003 | 0.006 | -0.010 | 0.014 |  |
| C2（Ind1-Ind3） | 0.001 | 0.002 | -0.003 | 0.005 |  |
| C3（Ind2-Ind3） | -0.001 | 0.006 | -0.012 | 0.010 |  |
